# Contamination of histology biopsy specimen - a potential source of error for surgeons: a case report

**DOI:** 10.4076/1757-1626-2-7619

**Published:** 2009-09-09

**Authors:** Neil G Burke, D McCaffrey, E Mackle

**Affiliations:** 1 Department of General Surgery, Craigavon Area Hospital, 68 Lurgan Road, Portadown, Northern Ireland, BT63 5QQ, UK

## Abstract

Tissue contamination is a common occurrence in pathology, but surgeons are relatively unaware of this. We present the case of a 45-year-old man with Barrett's oesophagus, in which the histology of routine biopsies of an asymptomatic patient, were reported as 'carcinoma in situ'. Further biopsies were taken over a three month period but showed no evidence of malignancy. Tissue contamination or 'cross over' was identified as the likely cause of the abnormal result. This case report highlights the importance of the correlation of the clinical and histopathological findings and tissue contamination should be considered when both of these findings are not consistent.

## Case presentation

A 45-year-old Caucasian man had a routine oesophagogastroduodenoscopy (OGD) showing a mild patchy area of possible Barrett's near the oesophago-gastric junction, and this was biopsied. He did not complain of dysphagia or weight loss. The histopathology results from the biopsy showed a small focus of tumour cells consistent in appearance with a carcinoma in-situ. Other tissue biopsies taken at the time showed Barrett's metaplasia only.

It was thought by histopathology that the focus of tumour could be a contaminant from another specimen. Possible sources of 'carry-over' at the time endoscopy by the surgeon, or during surgical gross dissection and also slide preparation were investigated, but no other specimens prepared had similar pathology. Repeat OGD with biopsies (×3) over a three month period showed there was no evidence of malignancy.

The abnormal and most recent specimens underwent DNA-PCR analysis which proved inconclusive due to insufficient DNA sample count. The hypothesis of tissue specimen contamination or cross-over remains the most likely cause of the abnormal result, in which the patient's benign tissue was contaminated by another cancerous tissue specimen.

## Discussion

The recognition of the discrepancy between the clinical history, endoscopic and histopathological findings was imperative in this case, otherwise this may have resulted in unnecessary major surgery for the patient.

Tissue specimen mix ups or 'carry-over' are a challenging problem in surgical pathology practice [1]. Surgeons should be aware that this a recurring problem in practice [2], and similar cases have occurred [3]-[5]. The reported rates of occurrence of contaminant tissues or cells have ranged from 0 to 8.8% (including prospective and retrospective cases) [6].

Tissue carryover can be produced during gross dissection of specimens, preparation of paraffin tissue blocks, during cutting of tissue sections and preparation of microscope slides [3]. The prevention of tissue contamination is, therefore, very difficult to avoid as it can occur at many different stages in the surgical or pathological preparation of the tissue sample [7].

Specimens should undergo DNA-based PCR techniques when there is suspected crossover involving similar tissue types and no obvious source of contamination. It has been shown to determine whether tissue contamination with another specimen has occurred [6]. However, this technique is expensive and there are limitations, as shown by this case, due to the small amount of tissue available from which DNA can be isolated or from degradation due to the fixation agent (formalin) [8].

Mitochondrial DNA haplotyping has also been used to exclude of the possibility of carry-over artefacts. Mitochondrial genetic typing is recommended for tissue samples with low DNA content and high degradation [3]. This was not performed on any of the specimens.

This case highlights the importance of the histopathology result being consistent with the clinical history and examination. In the event of a suspicious result, tissue contamination should be considered as a possibility after further negative biopies. Hence, further analysis of the biopsy specimen with DNA-PCR is essential, as the management for the patient may differ significantly.

## Abbreviations

DNA: deoxyribonucleic acid; OGD: oesophagogastroduodenoscopy; PCR: polymerase chain reaction.

## Consent

Written informed consent was obtained from the patient for publication of this case report and accompanying images. A copy of the written consent is available for review by the journal's Editor-in-Chief.

## Competing interests

The authors declare that they have no competing interests.

## Authors' contributions

NB was the main author, who wrote the paper and performed the literature research. DM aided with the discussion and the literature research. EM is the patient's consultant, and senior author of this article.
